# Knowledge, Attitude, and Practice of Organ Donation Among the Population of Al-Majma'ah Region, Saudi Arabia

**DOI:** 10.7759/cureus.59414

**Published:** 2024-04-30

**Authors:** Ahmed H Almutairi, Sultan A Alhassan, Saud A Alrajeh, Salem B Alqhatani, Ali S Alqarni, Mohammed Abdulaziz M Aljthalin, Yazid M Alghannam, Saud S Alanazi, Abdulaziz Munawir A Aldhafeeri, Faisal M Aldhafeeri, Nawaf H Alrumaih, Faisal M Alowain

**Affiliations:** 1 Emergency Medicine, Majmaah University, Al Majma'ah, SAU; 2 College of Medicine, Majmaah University, Al Majma'ah, SAU

**Keywords:** al-majma'ah region, organ donation, practice, attitude, knowledge

## Abstract

Background: Organ donation plays a pivotal role in addressing the global demand for transplantable organs and saving lives. The success of organ transplantation relies not only on medical advancements but also on the willingness of communities to participate in organ donation programs. In Saudi Arabia, specifically within the Al-Majma'ah region, understanding the dynamics of knowledge, attitudes, and practices related to organ donation is crucial for promoting a sustainable and ethical organ donation system.

Methods: A cross-sectional, retrospective study was utilized in this research, employing data from a sample of 564 participants from the general population of the Al-Majma'ah region, Saudi Arabia. The participants completed a self-administered questionnaire and ensured anonymity.

Results: About 545 (96.6%) respondents were familiar with the concept of organ donation, and 455 (80.7%) participants recognized the necessity for the blood groups of the donor and recipient to match before the transplant process. About 412 (73.0%) participants agreed with the practice of organ donation with 326 (57.8%) expressing support for the practice. About 417 (73.9%) participants reported that their religion permits or endorses organ donation/transplantation. A total of 151 individuals (26.8%) had a low knowledge level, with total scores below 50% (6 or lower). In contrast, 280 people (4.7%) demonstrated a moderate level of knowledge (scoring between 50% and 75%) (7 to 9). Additionally, 133 individuals (23.5%) showcased a high level of knowledge, with scores exceeding 75% (10 or higher). The study established a statistically significant association between age, marital status with p-values < 0.05 (0.001*), and the knowledge score toward organ donation. However, gender and monthly household income were not significantly associated with knowledge score toward organ transplant with p-values (p-value > 0.05).

Conclusion: The research findings indicated a moderate level of knowledge and a positive attitude toward organ donation among the general population of the Al-Majma'ah region in Saudi Arabia. Age and marital status were found to be significantly associated with the knowledge score toward organ donation. The study noted the desire and willingness to save lives through organ donation by the residents of the Al-Majma'ah region in Saudi Arabia.

## Introduction

Organ transplantation is one of the achievements of modern medicine, necessitating a reliance on medical technologies for the recipient. However, without organ donation, transplantation programs cannot exist. The effectiveness of any donation procedure is dependent on a multidisciplinary team of healthcare professionals working as a whole to ensure that organ donation is presented as an end-of-life care option and, when feasible, the shared goal of successful organ recovery and transplantation is achieved [1]. However, despite these advancements, creating a successful organ donation and transplantation program that effectively meets the population's needs is a complex challenge full of obstacles. While having knowledgeable and skilled medical professionals and managers, along with access to suitable facilities, is crucial, that alone is not enough for success. Various other factors must be considered, including obtaining consistent government backing, investing in sufficient infrastructure, implementing carefully designed reimbursement systems, and ensuring a highly trained and motivated workforce in adequate numbers [2].

Organ transplantation enhances patient survival and quality of life, and it significantly impacts public health and the socioeconomic burden of organ failure positively [3]. Organ transplantation is the only treatment for enhancing or preserving the life of patients with end-stage organ failure [4]. As per the World Transplant Registry, 56,116 European patients were awaiting a transplant at the end of 2014, starkly highlighting the substantial imbalance between supply and demand [4].

Countries with established donation and transplantation programs concentrate strategies in three areas where donation can be increased: expanding the "donor pool" such as through donation after circulatory death (DCD) and widening medical suitability criteria. These largely depend on transplantation practice and the capacity to achieve acceptable transplant outcomes, ensure all potential donors are identified and receive optimal medical management, and increase rates of consent [5]. As the total number of solid-organ transplants grows each year, the gap in organ supply is expanding. While many developed countries have implemented mandatory systems of registration for donation and transplant procedures, this is absent in many other countries, with a single comprehensive system to gather donation and transplant data on a common platform not in place [6].

As transplant outcomes have improved over the past three decades, more patients with end-stage kidney disease have chosen a transplant; however, there has not been a corresponding increase in the number of available organs [7]. As a result, the number of people on the waiting list, the time from listing to transplant, and the mortality while waiting continue to increase [7].

The scarcity of transplantable organs is an ongoing and challenging issue. Two primary factors contribute to the severe shortage of viable solid organs for transplantation in the United States. First, reliance on donations from deceased, brain-dead donors can only provide a limited number of potential donors; it has been estimated that no more than 15,000 such donors are available each year. Second, the level of agreement from next of kin regarding organ donation has restricted the pool of available organs for transplantation. On average, no more than 50% of those families approached for donation agree to donate [8].

## Materials and methods

Study design and setting

This study is descriptive and cross-sectional in nature. Before the main study, a pilot study was conducted to test the effectiveness and feasibility of the questionnaire. The research centers on the preliminary validation and enhancement of the survey instrument via a limited-scale survey [7]. The questionnaire was designed following a thorough review of existing literature concerning knowledge, attitude, and practice of organ donation. Twenty-five participants were enlisted to evaluate the questionnaire items for clarity, comprehensibility, and relevance to ensure their effectiveness in gathering the desired information. Following the pilot survey, initial data was scrutinized to pinpoint potential issues and identify areas for enhancement. The insights gained from this pilot study contributed to the refinement of the questionnaire for its eventual deployment in a broader study to assess knowledge, attitude, and practice of organ donation among the population of the Al-Majma'ah region, Saudi Arabia.

Sampling

The sample size was determined using the following formula:

$n = \frac{z^{2}\times pq}{d^{2}}$, where n: sample size; Z: standard deviation = 1.96; p: prevalence = 0.5; q: 1-p; DE: Design effect = 2; and d: error accepted = 0.05.

The sample size was determined to be 384 participants, and they were selected using the non-probability convenience sampling technique.

Data collection

The data was gathered through a pre-tested, self-administered questionnaire. Subsequently, the data analysis was conducted using the Statistical Package for the Social Sciences (SPSS) version 26 (IBM Corp., Armonk, NY). The questions included information on age, gender, knowledge, attitude, and practice of organ donation.

Inclusion and exclusion criteria

We included individuals aged 18 years and older, both Saudi and non-Saudi nationals, who were mentally competent and residing in the Al-Majma'ah region. However, individuals under 18 years of age were excluded from the study.

Data analysis

Statistical analysis was conducted using SPSS Statistics version 26. Descriptive statistics such as frequency and percentages were used for categorical variables, while mean and standard deviation were used for continuous variables. The Chi-squared test, t-test, and analysis of variance (ANOVA) were employed to assess differences and relationships between variables. A significance threshold (p-value) of 0.05 with a 95% confidence interval (CI) was utilized for hypothesis testing.

## Results

Table 1 presents sociodemographic information of the participants. The majority (383, 67.9%) of the participants were females with slightly more than half (288, 51.1%) of them aged 18-25 years old. More than half (331, 58.7%) of the participants were singles with a notable proportion (171, 30.3%) of them having a monthly household income of above 15,000 Saudi Riyals.

**Table 1:** Sociodemographic variables of the respondents Sociodemographic variables are presented in frequencies (n) and proportion (%).

Variables	Category	Frequency (Percentage)
Gender	Female	383 (67.9%)
	Male	181 (32.1%)
Age	Above 45 years	92 (16.3%)
	From 18 to 25 years old	288 (51.1%)
	From 26 to 35 years old	110 (19.5%)
	From 36 to 45 years old	74 (13.1%)
Marital status	Divorced	8 (1.4%)
	Married	220 (39.0%)
	Single	331 (58.7%)
	Widow	5 (0.9%)
Monthly household income	Above 15,000 Saudi Riyals	171 (30.3%)
	Below 5000 Saudi Riyals	129 (22.9%)
	From 10,000 to 14,999	110 (19.5%)
	From 5000 to 9999 Saudi Riyals	154 (27.3%)

Table 2 presents the participants’ knowledge about organ donation and transportation. The majority (545, 96.6%) of the respondents were familiar with the concept of organ donation. Interestingly, only 255 (45.2%) were informed about the Human Organ Transplantation Act, and even fewer (240, 42.6%) knew where to acquire organ donation cards. Most (399, 70.7%) of the participants recognized the possibility of donating organs from brain-dead patients with 337 (59.8%) of them citing the possibility of a certified brain-dead organ donor promptly getting disconnected from ventilator support. A significant majority, 455 (80.7%), recognize the necessity for the blood groups of the donor and recipient to match before the transplant process; with 418 (74.1%) of them noting the need for human leukocyte antigen of the donor to be identical to that of the recipient for any organ transplantation.

**Table 2:** Knowledge about organ donation Knowledge about organ donation is presented in frequencies (n) and proportion (%).

Statements	Frequency (Percentage)	
	Yes	No
I am familiar with the concept of organ donation.	545 (96.6%)	19 (3.4%)
Have come across the term organ transplantation.	522 (92.6%)	42 (7.4%)
I am informed about the Human Organ Transplantation Act.	255 (45.2%)	309 (54.8%)
I know where to acquire organ donation cards.	240 (42.6%)	324 (57.4%)
It is possible to donate organs from a patient declared brain-dead.	399 (70.7%)	165 (29.3%)
A certified brain-dead organ donor can be promptly disconnected from ventilator support.	337 (59.8%)	227 (40.2%)
Parents or guardians can make decisions on behalf of mentally disabled individuals regarding organ donation.	356 (63.1%)	208 (36.9%)
The blood groups of the donor and recipient must match.	455 (80.7%)	109 (19.3%)
The human leukocyte antigen of the donor must be identical to that of the recipient for any organ transplantation.	418 (74.1%)	146 (25.9%)
Carriers of Hepatitis B and C can donate all solid organs except the liver.	257 (45.6%)	307 (54.4%)
Malignancy is a contraindication of cadaveric organ donation.	303 (53.7%)	261 (46.3%)
All transplant recipients face an increased risk of opportunistic infections as a common complication.	397 (70.4%)	167 (29.6%)
Organ transplant recipients are more susceptible to developing cancer after the transplantation.	262 (46.5%)	302 (53.5%)

Table 3 presents participants’ attitudes toward organ donation. A significant majority of the participants expressed favor (412, 73.0%) and support (326, 57.8%) for organ donation. More than half (324, 57.4%) of the participants expressed their willingness to organ donation upon death. Regarding religious support, most (417, 73.9%) of the respondents reported that their religion permitted or endorsed organ donation/transplantation. Furthermore, a substantial proportion (348, 61.7%) of them did not hold the belief that their bodies should remain intact after death. Although 298 (52.8%) participants were worried about the premature termination of medical treatment for registered organ donors, more than half (305, 54.1%) of them believed that live organ donation was a more effective solution to the shortage than cadaveric organ donation.

**Table 3:** Attitude toward organ donation Attitude toward organ donation is presented in frequencies (n) and proportion (%).

Statements	Frequency (Percentage)	
	Yes	No
I am in favor of organ donation.	412 (73.0%)	152 (27.0%)
I am comfortable contemplating or discussing organ donation.	326 (57.8%)	238 (42.2%)
I am willing to donate my organs upon death.	324 (57.4%)	240 (42.6%)
I would consent to the donation of my family member's organs.	248 (44.0%)	316 (56.0%)
Organ donation is supported by my family.	247 (43.8%)	317 (56.2)
I believe donating my organs adds meaning to someone’s life.	417 (73.9%)	147 (26.1%)
My religion permits or endorses organ donation/transplantation.	417 (73.9%)	147 (26.1%)
I hold the belief that my body should remain intact after death.	216 (38.3%)	348 (61.7%)
I am afraid of disfigurement after I donate my organs.	290 (51.4%)	274 (48.6%)
I am worried about the premature termination of medical treatment for registered organ donors.	298 (52.8%)	266 (47.2%)
Live organ donation is a more effective solution to the shortage than cadaveric organ donation.	305 (54.1%)	259 (45.9%)

Table 4 shows the practice of organ donation. Nearly one-quarter (132, 23.4%) of the participants expressed their commitment and intention to donate an organ. Only 85 (15.1%) respondents had participated in the act of donating an organ. Regarding organ transplantation, the majority (479, 84.9%) had never undergone or received an organ for transplantation.

**Table 4:** Practice of organ donation Practice of organ donation is presented in frequencies (n) and proportion (%).

Statements	Frequency (Percentage)	
	Yes	No
I have committed or officially declared my intention to donate an organ.	132 (23.4%)	432 (76.6%)
I have participated in the act of donating an organ.	85 (15.1%)	479 (84.9%)
I have received an organ for transplantation.	85 (15.1%)	479 (84.9%)

Table 5 depicts the participants’ knowledge level score toward organ donation. The range of knowledge scores varied from a minimum of 0 to a maximum of 13 in the study, and the mean was 7.72 + 2.46. A total of 151 (26.8%) individuals had a low knowledge level; 280 (49.7%) of them demonstrated a moderate level of knowledge, while 133 (23.5%) of them demonstrated a high level of knowledge about organ donation.

**Table 5:** Participants’ knowledge level scores of organ donation Participants’ knowledge level scores of organ donation are presented in frequencies (n) and proportion (%).

Knowledge level	Total scores (%)	Total scores (n)	Frequency (Percentage)
Low knowledge level	Less than 50%	Score of 6 and less	151 (26.8%)
Moderate knowledge level	Between 50% and 75%	Score of between 7 and 9	280 (49.7%)
High knowledge level	Higher than 75%	Score of 10 and higher	133 (23.5%)

Table 6 provides the relationship between sociodemographic information and the knowledge score toward organ donation among the participants. The study established a statistically significant association between age, marital status with p-values < 0.05 (0.001*), and the knowledge score toward organ donation. Conversely, there was no statistically significant association between gender, monthly household income, and the knowledge score toward organ transplant with p-values > 0.05. The study noted that participants aged 18-25 years exhibited a higher knowledge score compared to older age groups (p < 0.001*). It was observed that people who were single and divorced had a higher knowledge score compared to being married and widowed. The analysis revealed that people who earned a monthly household income of less than 5000 Saudi Riyals had a higher knowledge score than those earning a higher monthly household income. However, the association was not statistically associated with the knowledge score toward organ donation.

**Table 6:** The association between sociodemographic information and the knowledge score toward organ donation * Significant at P < 0.05.

Variables	Category	Mean	Standard deviation	P-value
Gender	Female	7.13	2.19	0.472
	Male	8.24	2.29	
Age	Above 45 years	7.21	2.76	<0.001*
	From 18 to 25 years old	8.53	2.11	
	From 26 to 35 years old	7.72	2.65	
	From 36 to 45 years old	7.36	2.55	
Marital status	Divorced	8.22	2.45	<0.001*
	Married	6.67	2.78	
	Single	8.64	2.39	
	Widow	7.39	2.41	
Monthly household income	Above 15,000 Saudi Riyals	7.84	2.66	0.534
	Below 5000 Saudi Riyals	8.31	2.14	
	From 10,000 to 14,999	7.21	2.59	
	From 5000 to 9999 Saudi Riyals	7.59	2.41	

## Discussion

The study interrogated the knowledge, attitude, and practice of organ donation among the population of Al-Majma'ah region, Saudi Arabia. The majority of the participants were young adults in the age range of 18-25 years with a predominance of women. The study revealed that the vast majority were aware and familiar with the concept of organ donation and organ transplantation. The findings are consistent with a study conducted in the central region of Saudi Arabia by Almutairi, which revealed that the majority of the participants were aware of the concept of organ donation [9]. Similarly, the study conducted in Al-Kharj in Saudi Arabia by Agrawal et al. noted that the majority of the adult population had come across the term organ donation and were aware of the concept of organ transplantation [10]. The findings revealed that while a majority of the participants were aware of organ donation, only less than half of the participants were informed about the Human Transplantation Act, and even fewer knew where to acquire organ donation cards. A comparable study conducted by AlHarthi and Alzahrany in Taif, Saudi Arabia, found that more than half of the participants were aware of the organ donation centers but were not aware of their regulations [11]. These findings underscore the need for a concerted effort to inform the public about the policies and procedures of organ donation [12]. Most of the participants recognized the possibility of donating organs from brain-dead patients, with a significant number of them agreeing that a certified brain-dead organ donor can be promptly disconnected from ventilator support. The findings are incongruent with those of a study conducted in Eastern Morocco by Haddiya et al., which revealed that the majority of the surveyed population was aware that organ donations can come from both living donors and cadavers [13]. The current study noted that most of the participants recognize the necessity for the blood groups of the donor and recipient to match before the transplant process and that the human leukocyte antigen of the donor must be identical to that of the recipient for any organ transplantation.

The study results showed a moderate level of knowledge of organ donation, in which 47.9% scored 7-9 out of 13 points (mean of 7.72 and SD 2.46). This finding can be explained by the fact that the majority of the surveyed participants were young adults; most of them had attended higher institutions of learning where access to information is readily available [14]. Regarding the attitude, a notable proportion showed a positive attitude toward organ donation. More than half of the participants agreed with the practice of organ donation with a substantial proportion expressing support for the practice. Furthermore, a notable proportion of the participants were willing to donate their organs upon death. This is consistent with the findings of the study by Khushaim et al. among the general population of Jeddah in Saudi Arabia, which revealed that more than half of the participants were willing to donate their organs after death [15]. Similarly, in the study conducted in Nigeria by Ibrahim and Randhawa, nearly half of the participants expressed a desire for organ donation upon their death [16]. Regarding religious support, the majority of the respondents reported that their religion permits or endorses organ donation/transplantation. Additionally, more than half of them did not hold the belief that their bodies should remain intact after death. Although most of the Saudis are Muslim, the current study revealed that only a few people viewed religion as a barrier to organ donation and expressed unwillingness to donate on that account. This is in agreement with the findings by Islam, which noted that the Islamic religion encourages people to save lives and support each other as stated in Sunna and the Holy Quran [17]. In terms of the practice of organ donation, a notable proportion of the participants expressed their commitment and intention to donate an organ. Only a small proportion of the respondents had participated in the act of donating an organ.

The significant limitation in this investigation was the employment of a cross-sectional study design, which can only establish the relations between factors but not causalities. Additionally, the absence of some important sociodemographic information particularly on the education level of the participants limits the researchers' understanding of the association between the education level and the knowledge level about organ donation. Since the study was conducted in one region (Al-Majma'ah), the outcome cannot be generalized to the entire Saudi Arabia population.

## Conclusions

The study revealed a moderate level of knowledge and a positive attitude toward organ donation among the general population of the Al-Majma'ah region in Saudi Arabia. Age and marital status were found to be significantly associated with the knowledge score toward organ donation. The Islamic religion was found to be a significant inspiration for encouraging people to save lives and support others and not a barrier to organ donation. The study noted the desire and willingness to save lives through organ donation by the residents of the Al-Majma'ah region in Saudi Arabia. There is a need for increased public awareness about the importance of organ donation in saving lives, enhancing quality of life, and promoting sustainable and ethical organ transplantation systems among the general population in Saudi Arabia.
